# Detecting functional connectivity change points for single-subject fMRI data

**DOI:** 10.3389/fncom.2013.00143

**Published:** 2013-10-30

**Authors:** Ivor Cribben, Tor D. Wager, Martin A. Lindquist

**Affiliations:** ^1^Department of Finance and Statistical Analysis, Alberta School of Business, University of AlbertaEdmonton, AB, Canada; ^2^Department of Psychology and Neuroscience, University of ColoradoBoulder, CO, USA; ^3^Department of Biostatistics, Johns Hopkins Bloomberg School of Public HealthBaltimore, MD, USA

**Keywords:** functional connectivity, graph based change point detection, network change points, graphical lasso, stability selection, stationary bootstrap, dynamic connectivity

## Abstract

Recently in functional magnetic resonance imaging (fMRI) studies there has been an increased interest in understanding the dynamic manner in which brain regions communicate with one another, as subjects perform a set of experimental tasks or as their psychological state changes. Dynamic Connectivity Regression (DCR) is a data-driven technique used for detecting temporal change points in functional connectivity between brain regions where the number and location of the change points are unknown *a priori*. After finding the change points, DCR estimates a graph or set of relationships between the brain regions for data that falls between pairs of change points. In previous work, the method was predominantly validated using multi-subject data. In this paper, we concentrate on single-subject data and introduce a new DCR algorithm. The new algorithm increases accuracy for individual subject data with a small number of observations and reduces the number of false positives in the estimated undirected graphs. We also introduce a new Likelihood Ratio test for comparing sparse graphs across (or within) subjects; thus allowing us to determine whether data should be combined across subjects. We perform an extensive simulation analysis on vector autoregression (VAR) data as well as to an fMRI data set from a study (*n* = 23) of a state anxiety induction using a socially evaluative threat challenge. The focus on single-subject data allows us to study the variation between individuals and may provide us with a deeper knowledge of the workings of the brain.

## 1. Introduction

Traditionally, functional magnetic resonance imaging (fMRI) studies have concentrated on locating brain regions showing task-related changes in neural activity, for example, greater activity during an experimental task than during a baseline state (Lindquist, 2008). The voxel-wise general linear model (GLM) (Worsley and Friston, 1998) has become the standard approach for analyzing such data. However, in order to gain a deeper insight into the neural processing of the brain as the subject performs a set of experimental tasks or changes psychological state, there is now an intense interest in the field to study the interactions or connectivity between distinct brain regions.

A very active area of neuroimaging research involves examining the directed (effective connectivity) and undirected association (functional connectivity), between two or more spatially remote brain regions (Friston et al., 1993; Biswal et al., 1995). For example, the “Human Connectome” project is currently uncovering the structural and functional determinants of brain activity using information from anatomical, functional and effective connectivity (Sporns et al., 2005). For fMRI data, the covariance or correlation matrix as well as the precision matrix (inverse covariance matrix) can be estimated and used to measure functional connectivity between different regions based on their time series. However, these techniques are only useful when estimating static functional connectivity networks or when the experimental condition does not change over time. The techniques do not consider the possibility of changing functional connectivity or the dynamical nature of interactions between brain regions. If the dynamic connectivity (or dependencies) between brain regions are not taken into account, the true dynamics are not captured and the overall connectivity structure of the data will simply represent an aggregate of all the changing connectivities between runs. Specifically, the distinction between the different psychological states would not be found, rather an average of the functional connectivity would be estimated, which in many experiments would lead to meaningless results.

The popular PPI (PsychoPhysiological Interactions; Friston et al., 1997) method addresses the dynamic nature of functional connectivity but it assumes the timing of the various contexts or state changes is known. However, often, it is difficult to specify the nature, timing and duration of the psychological processes being studied *a priori*. Chang and Glover (2010) and Allen et al. (2013) introduced methods for estimating time varying connectivity between brain regions for resting state data using a moving window. Hence, an important extension of these works is to have methods that can detect changes in connectivity, regardless of the nature of the experimental design. In order to find evidence of fluctuations in functional connectivity over the course of the experiment, we previously introduced a technique called Dynamic Connectivity Regression (DCR; Cribben, 2012; Cribben et al., 2012). DCR is a data-driven method for detecting functional connectivity change points between brain regions where the number of change points and their location are not known in advance. After finding the change points, the method estimates a graph or series of relationships between the brain regions for data in the temporal partition that falls between pairs of adjacent change points. It is assumed that the graph does not change within each partition. The motivation behind the method stems from the fact that under certain circumstances the dynamic manner in which the brain regions interact with one another over time is of primary interest. DCR utilizes the graphical lasso or glasso (Friedman et al., 2007) and so it allows for change point and graph estimation in a high-dimensional context by setting many elements of the precision matrix to zero. DCR can be applied to estimate the dynamic interaction between the brain regions as well as the timing, duration and details of the interaction.

In Cribben et al. (2012), DCR was used to estimate change points and undirected graphs for multi-subject data. In this paper, we focus exclusively on single-subject data because group average analyses risk removing unique individual patterns of activity and analyzing single-subject data allows us to understand the sources of intersubject variability in brain activity. It is assumed that by averaging across the group, the noise effects are removed but by analyzing individual subjects and the variations between them, the results may provide insight to the understanding of the mind/brain relationship. In other words, while analyses of multi-subject data are highly valuable for understanding general cognitive processes, the study of variation between individuals may be able to provide us with a deeper knowledge of the workings of the brain. Furthermore, by analyzing multi-subject data we implicitly average connectivity patterns across the group, which may lead to meaningless results. An example of this can be found in section 5, where subjects with positive connectivity is canceled out by subjects with negative connectivity. Evaluating how functional brain networks change over time and relating this information to brain dysfunction promises to be very important for understanding the underlying mechanisms of brain disorders such as depression and Alzheimer's disease.

To this end, we introduce a new DCR algorithm that involves the addition of extra steps to the old algorithm to increase accuracy for single-subject data with a small number of time points. The extra steps prove vital in situations where there are small and subtle connectivity changes. We show that the new DCR algorithm is very adept at finding both major and minor changes in the functional connectivity structure. A comparative study between the old and new algorithm is carried out in the simulation study. The stationary bootstrap inferential procedure for detecting significant change points is also revised accordingly to obtain more accurate estimates of the distribution of the BIC reduction at each candidate change point. The glasso technique for estimating sparse graphs can be severely impacted by the inclusion of only a few contaminated values, such as spikes that commonly occur in fMRI time series, and the resulting graph has the potential to contain false positive edges (Finegold and Drton, 2011). The second extension involves performing a bootstrap inferential procedure on the edges or partial correlations in the undirected graphs to remedy the issue of the false positive edges.

By carrying out the analysis on individual subjects we do not need to collapse information across subjects or assume that the graph structure between each pair of change points is the same across subjects. The subject's change points and partition specific undirected graphs are thereafter compared to determine whether the change points and connectivity structures are common across all subjects. We also introduce a new Likelihood Ratio test on the similarity of the sparse precision matrices across subjects. With this information we can make inferences about whether the assumption of equal connectivity structure across subjects is valid. The matter of whether to combine information across subjects (to analyse group average results) or whether to look at individual patterns of brain activity is critical for individual treatment of brain disorders and very topical in the field of neuroscience (see Van Horn et al., 2008 for a discussion). As noted in Varoquaux et al. (2010), the use of multiple datasets is very challenging due to the variability between subjects coupled with the variability of functional signals between experimental runs.

This paper is organized as follows. In section 2 we begin by briefly discussing the theoretical foundations of DCR. Next we describe the new DCR algorithm for single-subject data with a small number of time points and inferential procedures for determining statistically significant connectivity change points and statistically significant edges in the undirected graphs. We also discuss a Likelihood Ratio test for comparing precision matrices across and within subjects. In sections 3 and 4 we outline the simulations and the fMRI study that the new DCR is applied to respectively. We describe the results of the simulations and experimental data in section 5. The results display DCR's capability to find connectivity change points for single-subject data with only a limited number of observations thus allowing for comparison across subjects.

## 2. Materials and methods

### 2.1. Dynamic connectivity regression (DCR)

A more detailed explanation of the theory behind DCR can be found in Cribben et al. (2012) and in Cribben (2012). Suppose **Y** (e.g., several brain regions) has *n* i.i.d data points sampled from a zero mean *p*-dimensional multivariate Gaussian distribution, *N*(**0**, Σ). In order to estimate the dependence structure of **Y** we could use the sample covariance matrix, $\hat{\Sigma }={\left(n-1\right)}^{-1}\sum_{i=1}^{n}\left(y_{i}-\bar{y}\right){\left(y_{i}-\bar{y}\right)}^{T}$ (estimate of Σ). Alternatively we could use the precision matrix (inverse covariance matrix), Ω = Σ^−1^. The natural estimator for Ω is ${\hat{\Sigma }}^{-1}$. However, we could also consider regularization and sparse techniques which are especially needed when *p* > *n*. The most widely used precision matrix estimation procedures are based on penalized likelihood maximization or constrained error minimization approaches which are optimization algorithms with different objective functions. All of these estimation procedures assume that the dependence structure between the data remains constant. However, in many applications, including neuroimaging, the dependency structure between the individual voxel or ROI time series varies over time. If the dynamic dependencies are not properly accounted for, the overall dependence structure will simply be an aggregate of all the different dependency structures. DCR detects change points in functional connectivity between brain regions, without knowledge on the number or location of the change points, and estimates a graph or set of relationships between the brain regions for data between each pair of change points.

DCR builds upon the extensive literature on graphical models (Whittaker, 1990; Edwards, 1995; Cox and Wermuth, 1996). Within this framework, graphical models display the dependency structure of a random vector **Y** using a graph G. Graphs are mathematical structures that can be used to model pair-wise relationships between variables. They consist of a set of vertices V and corresponding edges E connecting pairs of vertices. Here, we focus exclusively on undirected graphs which do not infer the directionality of dependence or functional connectivity between the variables or brain regions. A graph of **Y** can alternatively be represented using the precision matrix (inverse covariance matrix) of **Y**, with the elements of the matrix corresponding to edge weights. Here a missing edge between two vertices in the graph indicates conditional independence between brain regions, giving rise to a zero element in the precision matrix. Throughout this paper we will model dependencies between brain regions using the precision matrix or undirected graphs and we will use them interchangeably throughout the paper.

### 2.2. The new DCR algorithm

In this work, we introduce a new DCR algorithm necessary for the efficient and accurate analysis of single-subject fMRI data. The step-by-step description of the new algorithm can be found in section A.1. of the Appendix. The new steps involve recalculating the Bayesian Information Criterion (BIC: Equation 4) at each candidate change point after the first pass of the algorithm, altering the stationary bootstrap inference procedure on the candidate change points and including a new bootstrap inference procedure on the undirected graphs within each partition in order to remove the false postive edges prevalent in undirected graphs for single-subject data.

The objective of the algorithm is to split the time course or the time domain, X, into partitions χ_1_,…, χ_*m*_. As before, our greedy procedure begins by calculating the sample mean and sample covariance matrix for the entire data set using Equations (1, 2), respectively.

(1) $${\hat{\mu }}_{\chi_{j}}=\frac{1}{\sum_{i=1}^{t_{j}}I\left(x_{i}\in \chi_{j}\right)}\sum_{i=1}^{t_{j}}y_{i}I\left(x_{i}\in \chi_{j}\right)$$

(2) $${\hat{\Sigma }}_{\chi_{j}}=\frac{1}{\sum_{i=1}^{t_{j}}I\left(x_{i}\in \chi_{j}\right)}\sum_{i=1}^{t_{j}}\left(y_{i}-{\hat{\mu }}_{\chi_{j}}\right){\left(y_{i}-{\hat{\mu }}_{\chi_{j}}\right)}^{T}I\left(x_{i}\in \chi_{j}\right)$$

For the entire data set calculation χ_*j*_ is all the time points and *t*_*j*_ is the number of time points in the entire experiment. Estimates of the precision matrix, $\hat{\Omega }$, for the entire data set are then obtained using the graphical lasso or glasso (Friedman et al., 2007) for a specified full path of λ values using Equation (3). The *l*_1_-penalty in this equation induces sparsity and regularization on the elements of the estimated precision matrix.

(3) $$\hat{\Omega }={\text{argmin}}_{\Omega ≻0}\left\{\text{tr}\left(\hat{\Sigma }\Omega \right)-\text{log}|\Omega |+\lambda \|\Omega {\|}_{1}\right\}$$

The tuning parameter λ controls the sparsity of the precision matrix, large values giving rise to a very sparse precision matrix and small values giving rise to a very “full” precision matrix or graph. An efficient algorithm has been developed (Friedman et al., 2007) for finding $\hat{\Omega }$, an estimate of the precision matrix, that estimates a single row (and column) of Ω in each iteration by solving a lasso regression. We chose the value of λ that minimizes the BIC

(4) $$\text{BIC​}=\text{​}\sum_{i=1}^{t_{j}}\left[\left(\text{tr}\left[\Omega \left(y_{i}-\mu \right){\left(y_{i}-\mu \right)}^{T}\right]-\text{log}|\Omega |\right)\right]+k\cdot \text{log}\left(t_{j}\right)$$

where *t*_*j*_ is the number of data points in the partition and *k* is the number of off-diagonal elements in the precision matrix. After estimating the precision matrix based on BIC minimization and identifying non-zero edges, the model is refit without the *l*_1_-constraint while keeping the zero elements in the matrix fixed to reduce bias and improve the model selection performance (Liu et al., 2010).

The minimum BIC score for the entire data set is recorded, providing a baseline in which to evaluate subsequent splits. Note that the estimated precision matrix for the entire data set is simply an aggregate of all the changing connnections (or partial correlations) assuming there exists a dynamic connectivity structure in the data. The BIC is a model selection criterion based on combining the likelihood function with a penalty term that guards against over-fitting. Hence, it balances the dual needs of adequate model fit and model parsimony.

Upon completion of this step, the data is then partitioned into two sets; a left subset consisting of time points {1:Δ} and a right data set consisting of {Δ + 1:*T*}, where *T* represents the length of the time course. The choice of Δ is of particular importance as it also represents the minimum possible distance between adjacent change points and its value can be adjusted depending on the existence of *a priori* knowledge about the spacing of changes in functional connectivity. Unlike the multi-subject analysis presented in Cribben et al. (2012), where DCR could find change points with close proximity (i.e., with Δ = 10), the single-subject analysis in this paper requires Δ to be a minimum of 30–40 time points to ensure that there is enough data to provide stable estimates of the sample means and sample covariance matrices and hence the precision matrices. The sample mean and covariance matrices for both subsets are calculated separately using Equations (1, 2), respectively. Again the full regularization path of λ values for the glasso is run and the values of λ, and corresponding precision matrices, are chosen based on minimizing the BIC. Each model is then refit, as above, keeping the zero elements in each precision matrix fixed, and the combined BIC scores for the two subsets are recorded. This procedure is repeated along the entire time path, with the data partitioned into two subsets with split points ranging from {1:Δ + 1} to {1:*T* − Δ}. The partition with the smallest combined BIC score is chosen and, if its value is less than the BIC score for the entire data set, the corresponding time point is used to partition the data and the first candidate change point is identified.

The DCR procedure continues by recursively applying the same method to each individual partition element until they can no longer be split any further. In other words, if the first candidate change point occurs at time ρ, the procedure is repeated for both the data set consisting of time points {1:ρ} and the one consisting of time points {ρ + 1:T}. The procedure is repeated until no further splits reduce the BIC score.

Once the candidate change points have been found, a new extra refitting step is carried out to improve the estimation of the BIC reduction at each split. In other words, the first candidate change point separates the entire data set into two similar data sets based on their functional connectivity structure. But if further candidate change points are found then the first candidate change point's BIC reduction is inaccurate and requires reestimation. This is particularly important for single-subject experimental data sets with few time points. When the DCR algorithm recursive procedure is finished, the candidate change points are ordered in time. If the first candidate change point occurs at time point ρ and the second candidate change point occurs at time point ν, the BIC is calculated for data sets {1:ρ}, {ρ + 1:ν} and {1:ν} by estimating the sample means, sample covariance matrices and precision matrices along the full path of λ values for each partition. The new BIC reduction at time point ρ is then

(5) BIC{1:ν}−[BIC{1:ρ}+BIC{ρ+1:ν}]

By sequencing through all the candidate change points in this manner and recalculating the BIC reduction the model's accuracy is improved (compare Figures 4A,B as well as Figures 5A,B). This extra set of calculations only marginally increases the algorithm's computation time. After completion of the algorithm, the new DCR will have split the experimental time course into connected partitions χ_1_,…, χ_*m*_ and within each partition χ_*j*_, the glasso is again used to estimate an undirected graph *Ĝ_j_*.

The idea behind the DCR algorithm involves comparing the BIC of the entire data set with the BIC of two subsets (right and left) of the data. In the former case the model contains *k* parameters while in the latter case the model contains *k*_1_ + *k*_2_ parameters, where *k*_1_ and *k*_2_ represent the number of parameters in the left and right subset, respectively. This allows for more free parameters in the two subsets case. However, this bias is balanced out by the fact that the full data model contains *T* observations while the split data model contains some combination of data that sums to *T*. It is evident that the BIC penalizes the split data model more than the BIC of the entire data set as the goal is to minimize the BIC. Hence, false positive BIC reductions or false positive change points are not of great concern to the DCR algorithm. For more details see Cribben (2012).

### 2.3. Detecting significant change points

To determine whether significant change points exist, confidence bounds for each non-zero BIC reduction at each splitting time are created using a new stationary bootstrap procedure (Politis and Romano, 1994). The stationary bootstrap, unlike a permutation procedure, is mindful of the dependency structure inherent in the data where successive time points are assumed to be correlated, but observations “far apart” uncorrelated. The stationary bootstrap is an adaptation of the block bootstrap (Liu and Singh, 1992; resamples blocks of observations instead of individual observations) that allows for randomly varying block sizes. The new stationary bootstrap procedure is similar to the stationary bootstrap procedure introduced in the original DCR paper (Cribben et al., 2012), however, it is altered to reflect the changes in the DCR algorithm described above.

The new stationary bootstrap procedure begins by running the new DCR algorithm to determine all the ordered candidate change points. Assume the first and second ordered candidate change points are ρ and ν, respectively. To test whether the BIC reduction at change point ρ is significant, data within the partition {1:ν} is repeatedly resampled across time using the stationary bootstrap. For each resampling, the data is split into two parts, one consisting of time points {1:ρ} and the other of {ρ + 1:ν}. The combined BIC score from each subset is subtracted from the BIC of the entire resampled data set {1:ν} using the stationary bootstrap and the results are combined across replications to create a stationary bootstrap distribution for the BIC reduction at the candidate change point ρ. The procedure is repeated for each non-zero BIC reduction or candidate change point. For example, in order to check significance for the second candidate change point ν, data within the partiton {ρ + 1:ϵ}, where ϵ is the third candidate change point, is repeatedly resampled across time using the stationary bootstrap.

The (1 − α/2) and α/2 quantiles of the stationary bootstrap distribution of each non-zero BIC reduction for each splitting time can be plotted and interpreted as 100(1 − α)% confidence bounds. For a candidate change point at time ρ, if the BIC reduction for the original data is more extreme (either larger or smaller) than the 100(1 − α)% confidence bounds computed using repeated stationary bootstrap replicates, we conclude there is a significant change point at ρ, indicating a change in connectivity. This new stationary bootstrap procedure decreases the computation time of the algorithm as the size of the resampled data sets are now smaller.

### 2.4. Constructing undirected graphs

Once the significant change points have been found, the data is divided into partitions defined by the splits. For each partition, the sample mean and sample covariance matrix are estimate using Equations (1, 2), respectively. Using the full path of λ values in Equation (3), the optimal λ value and precision matrix is chosen based on minimizing the BIC. It is necessary to carry out these extra calculations as the sample means, sample covariance matrices, precision matrices, BIC reductions and λ values associated with each partition calculated using the new refitting scheme in the DCR algorithm assumes that all candidate change points are significant. If not, the estimated values associated with the partition are no longer valid as the time points in certain partitions change as a result.

In order to make the off-diagonal elements of the precision matrix, *P* = Ω, more interpretable they are converted into partial correlations, *r*_*ij*_, using the following formula

(6) $$r_{ij}=-\frac{p_{ij}}{\sqrt{p_{ii}p_{jj}}}$$

The undirected graphs in this work plot the partial correlations between the brain regions.

### 2.5. Testing edges

A shortcoming of the glasso is the excessive number of false positive edges present in the estimated undirected graphs. This fact is highlighted for multi-subject data in Cribben et al. (2012), but it is noteworthy that the false positive partial correlations tend to be small. The number of false positive edges increases in the single-subject setting, which is the emphasis here, with an excess of edges also complicating interpretation of the undirected graphs. Hence, we include an additional step in the new DCR algorithm by performing inference on the edges using information from a bootstrap procedure.

The procedure is carried out for each partition specific undirected graph, computed as described in section 2.4. For a partition χ_*j*_, a bootstrap data matrix Y^*^_χ*j*_ is formed by randomly selecting with replacement rows of data from the original partition specific data matrix **Y**_χ*j*_. We estimate a precision matrix (or undirected graph) for the new data set Y^*^_χ*j*_ for the full path of λ values and we choose λ and the undirected graph based on minimizing the BIC. We record each partial correlation in the undirected graph. A graph containing 20 vertices has 190 (0.5 × *p* × (*p* − 1)) possible edges or partial correlations. This procedure is repeated a large number of times (say, 1000). We then count the number of times glasso estimates the edge to be non-zero in the 1000 bootstrap runs. These proportions represent the probability of edge selection. For example, if glasso estimates the edge to be non-zero in 990 bootstrap runs, the edge has an estimated selection probability of 0.99. For each edge, if its selection probability is greater than some pre-defined threshold, say 0.95, then the edge or partial correlation is significant and it remains in the undirected graph. On the other hand, if its estimated selection probability is less than the pre-defined threshold then the edge or partial correlation is not significant and it is removed from the undirected graph. This procedure solves the problem of false positive edges in the undirected graphs estimated by the glasso and by extension DCR.

This bootstrap inferential procedure is similar to the subsampling stability selection approach of Meinshausen and Bühlmann (2010). The goal is to control the familywise type I multiple testing error in a high dimensional setting by looking at the selection probabilities of every variable (or edge) under subsampling. In their framework, the data are subsampled many times and they choose all variables that occur in a large fraction of the resulting selection sets. They retain variables with a high selection probability and remove those with low selection probabilities. The exact cutoff π_thr_ is a tuning parameter but they notice that the results do not vary much for sensible choices of it.

### 2.6. Comparing graphs

Usually in an fMRI study, several subjects are scanned. In the multi-subject DCR (Cribben et al., 2012), we assumed the connectivity between brain regions was similar for each subject. By combining information across subjects, the multi-subject DCR averages the connectivity patterns in the group of subjects as they perform a set of tasks or change psychological state. However, in many circumstances, this may not be wise as we risk losing the unique patterns of activity in the individual subjects. This paper shows that DCR can be run on all subjects individually and the change points and partition specific undirected graphs can be compared and tested across subjects. For example, in a task based fMRI experiment such as an ABAB design, we could check whether the connectivity in the first block is the same as the connectivity in the third block.

Massa et al. (2010) introduced a Likelihood Ratio test for testing the equality of two or more precision matrices or undirected graphs. The test originates from the test of equal covariance matrices for Gaussian graphical models (Anderson, 2003). It has the following setup. Suppose that random samples are taken from *s* multivariate normal (MVN) populations each with *p* variables **Y**_1_,…,**Y**_s_ and the *i*th population has the mean vectors **μ**_*i*_ and the covariance matrix Σ_*i*_. Assuming that the **μ**_*i*_s vary from population to population, the maximum likelihood estimators of these are the sample mean vectors. Assume also that the mean vectors are zero and we have (*y*^*j*^_1_), *j* = 1, …, *n*_1_ observations from *N*_*p*_(**0**, Σ_1_), (*y*^*j*^_2_), *j* = 1, …, *n*_2_ observations from *N*_*p*_(**0**, Σ_2_),…, and (*y*^*j*^_*n*_), *j* = 1, …, *n*_*s*_ observations from *N*_*p*_(**0**, Σ_*s*_). Let Σ^−1^_1_ = Ω_1_,…, Σ^−1^_*s*_ = Ω_*s*_. Under the null hypothesis of equal precision matrices, *H*_0_ : Ω_1_ = … = Ω_*s*_, the number of unknown parameters in the common precision matrix Ω_0_ is $\frac{1}{2}$*p*(*p* + 1). It can be shown that the maximum likelihood estimator of Ω_0_ is

(7) $${\hat{\Omega }}_{0}=\sum_{i=1}^{s}n_{i}{\hat{\Omega }}_{i}/n$$

where Ω_*i*_ is the *i*th sample precision matrix, *n*_*i*_ is the number of observations in the *i*th sample and *n* (= *n*_1_ + … + *n*_*s*_) is the total number of observations. The maximized log likelihood for this model is then

(8) $$l_{0}=K+\frac{1}{2}n\text{log}\left(\left|{\hat{\Omega }}_{0}\right|\right)$$

where *K* = −$\frac{1}{2}$*np*log(2π) − $\frac{1}{2}$*np* and |${\hat{\Omega }}_{0}$| is the determinant of matrix ${\hat{\Omega }}_{0}$. Under the alternative hypothesis (unequal precision matrices), the number of unknown parameters is $\frac{1}{2}$*sp*(*p* + 1). The maximized log likelihood for this model is then

(9) $$l_{1}=K+\sum_{i=1}^{s}\frac{1}{2}n_{i}\text{log}\left(\left|{\hat{\Omega }}_{i}\right|\right)$$

Assuming the null hypothesis (equal precision matrices) is correct, the LR test statistic is

(10) $$\begin{array}{l} -2\text{log}\Lambda =2\left(l_{1}-l_{0}\right)=n\text{log}\left(\left|{\hat{\Omega }}_{0}\right|\right)-\sum_{i=1}^{s}n_{i}\text{log}\left(\left|{\hat{\Omega }}_{0}\right|\right)\\ \text{​​​​​​​​​​​​​​​​ ​}=\sum_{i=1}^{s}n_{i}\text{log}\left(\frac{\left|{\hat{\Omega }}_{0}\right|}{\left|{\hat{\Omega }}_{j}\right|}\right) \end{array}$$

This test statistic has a χ-squared distribution with degrees of freedom equal to the number of estimated parameters under the alternative hypothesis minus the number of estimated parameters under the null hypothesis. However, the test statistics's asymptotic χ-squared distribution and its corresponding degrees of freedom are not valid for sparse or regularized precision matrices which is the emphasis of this paper. Hence, we introduce a new bootstrap LR test. It is set up as follows; suppose we have *s* subjects each with *p* variables **Y**_1_,…,**Y**_*s*_. Under the null hypothesis of equal precision matrices for all *s* subjects, we can bootstrap rows of data points across subjects. Thus, for each subject, we create a bootstrap data matrix Y^*^_i_ = **Y**_*i*, 1^*^_,…,**Y**_*i*, t^*^_ by randomly selecting with replacement rows of data from the original data matrices **Y**_1_ = (**Y**_1, 1_,…, **Y**_1, *t*_),…,**Y**_*s*_ = (**Y**_*s*, 1_,…,**Y**_*s, t*_). For each subject we estimate a precision matrix for its new bootstrap data set Y^*^_i_ for the full path of λ values. We choose λ and its corresponding precision matrix based on the minimum BIC. We then estimate the test statistic in Equation (10). This procedure is repeated a large number of times (say, 1000) to create a bootstrap LR distribution.

## 3. Simulations

In order to assess the performance of the new DCR algorithm and new inference procedures, a number of simulations are performed. In Cribben et al. (2012) the simulation study concentrated on multi-subject data but here we focus exclusively on single-subject data.

The first simulation illustrates the application of the new DCR algorithm to identically distributed data (i.e., null data). The next simulations illustrate the application of the new DCR to vector autoregression (VAR; Zellner, 1962; Hamilton, 1995) data. For applications to simulated Multivariate Normal data, please refer to Cribben (2012). VAR is an econometric model, generalizing the univariate AR model, commonly used to capture the evolution and the interdependencies between multiple time series. More details on VAR models are included in section A.2. of the Appendix. VAR data are representative of the properties underlying fMRI data in that they have the property of autocorrelation within the individual time series (brain regions) but can have the possibility of non-zero cross correlation and lagged cross correlation between the time series (brain regions). For each VAR simulation, for regions not apart of the connected system, their time series are made up of i.i.d Gaussian noise indicating a lack of connectivity.

The objective of each simulation is to find the times of the functional connectivity change points and to estimate the connectivity structure within each partition assuming that the location and number of change points are unknown. The stationary bootstrap (section 2.3) and bootstrap (section 2.5) procedures are used in order to perform meaningful inference procedures on the change points and on the edges (connections) between the brain regions, respectively.

## 4. Experimental data

We use the same data set here as in Cribben et al. (2012). The data was taken from an anxiety-inducing experiment (Lindquist and McKeague, 2009; Wager et al., 2009a,b). The experimental task was a variant of a well-studied laboratory paradigm for eliciting social threat, in which subjects are instructed to deliver a speech under evaluative pressure. The design was an off-on-off design, with an anxiety-provoking speech preparation task occurring between resting periods. Participants were informed that they were to be given 2 min to prepare a 7 min speech, and that the topic would be revealed to them during scanning. They were informed that after the scanning session they would deliver the speech to a panel of expert judges, though there was “a small chance” they would be randomly selected not to give the speech. After the start of fMRI acquisition, participants rested for 2 min by viewing a fixation cross. At the end of this period, participants viewed an instruction slide for 15 s that described the speech topic, which was “why you are a good friend.” The slide instructed participants to be sure to prepare enough for the entire 7 min period. After 2 min of silent preparation, another instruction screen appeared (a relief instruction, 15 s duration) that informed participants that they would not have to give the speech. An additional 2 min period of resting baseline completed the functional run.

Data was acquired and preprocessed as described in previous work (Wager et al., 2009a). During the course of the experiment a series of 215 images were acquired (TR = 2 s). In order to create ROIs, time series were averaged across the entire region. The data analyzed here consists of four ROIs and heart rate for *N* = 23 subjects (Figure 8A). The regions in the data set were chosen due to the fact that they showed a significant relationship to heart rate in an independent data set. The temporal resolution of the heart rate was 1 s compared to 2 s for the fMRI data. Hence, the heart rate was down-sampled by taking every other measurement.

The new DCR approach was carried out on all 23 subjects individually and we were interested in testing whether stressor onset was associated with changes in the connectivity between the brain regions and heart rate. Each subject's change points and partition-specific undirected graphs are compared in order to observe whether the change points and connectivity structures are common across subjects. We also perform the LR test on the similarity of the precision matrices across subjects. With this information we can make inferences about whether the assumption of the same connectivity structure across subjects is valid and helpful.

## 5. Results

For each simulation, in order to guarantee reasonable estimates of the sample mean vectors and sample covariance matrices, we set δ = 35, that is, we set the minimum distance between change points to be 35 data points. This length is necessary for the single-subject data unlike the multi-subject data simulations in Cribben et al. (2012). If δ < 35 false positive change points occur due to the instability of the mean and covariance estimates. For each simulation we plot the BIC reduction against time in order to find the significant change points. In these plots, the vertical black lines represent the BIC reduction at the particular time point and the red triangles in the simulated data sets represent the 0.975 and 0.025 empirical quantiles of the BIC reduction based on 1000 stationary bootstrap relications of the data for each time point with a non-zero BIC reduction. For the stationary bootstrap, we use ψ = 1/0.05, that is, the average block length is equal to 20 time points for a data set containing 100 time points. The new DCR algorithm stationary bootstraps the data within each partition after the candidate change point have been found so the average length of the block changes depending on the size of the partition. Also, for all simulations, the empirical quantile threshold for the bootstrap distribution of the edge values is 0.75 based on 1000 runs (stability selection; Meinshausen and Bühlmann, 2010). This cutoff value provided the right balance for including the correct edges and removing the false positive edges in the undirected graphs. The correct undirected graphs are also plotted for comparison purposes. The black and red edges in the undirected graphs represent positive and negative partial correlation, respectively. The thickness of the edges are directly related to the strength of the connectivity between two brain regions.

### 5.1. Simulation 1

**Figure 1:**
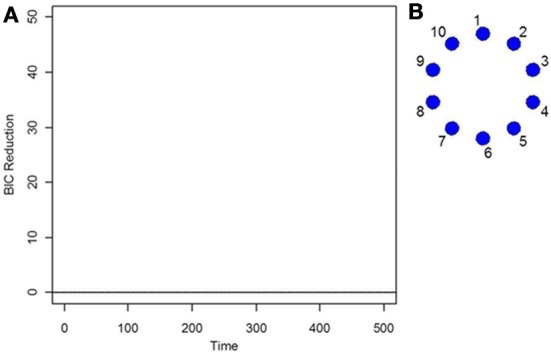
**(A)** The splitting times plotted against BIC reduction for the i.i.d data set (simulation 1) using the new DCR. **(B)** The corresponding undirected graph for this data set.

As the data is i.i.d with no functional connectivity between the brain regions, the new DCR correctly finds no BIC reduction for any time point (Figure 1A) and so the functional connectivity between the brain regions remains constant throughout the whole time series. As the data is white noise, the new DCR correctly finds no networks or functional connectivity between the brain regions (Figure 1B). DCR was also applied to the same simulated i.i.d data but with 10 spikes of magnitude 4 randomly added to the entire series. Again, it correctly finds no change points and no edges or functional connectivity between the time series (the figures are the same as Figures 1A,B and are not included).

### 5.2. Simulation 2

**Figure 2:**
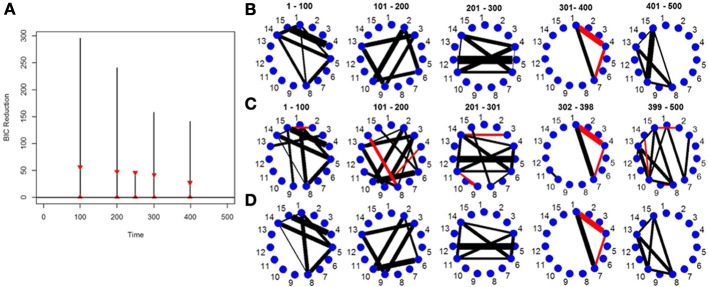
**(A)** The splitting times plotted against BIC reduction for the VAR data set with four connectivity change points (simulation 2) using the new DCR algorithm. The vertical black lines represent the BIC reduction at the particular time point and the red triangles represent the 0.975 and 0.025 empirical quantiles of the BIC reduction based on 1000 stationary bootstrap relications of the data for each split. **(B)** The true undirected graphs for each partition. The undirected graphs for this data set using the new DCR and **(C)** no inference on the edges and **(D)** inference on the edges.

Figure 2A depicts the splitting times plotted against BIC reduction for a VAR simulation with four connectivity change points. The new DCR correctly finds all four connectivity change points. Figure 2B shows the true partial correlations for this VAR data set for the correct change points. Figures 2C,D show the undirected graphs for the partitions found in Figure 2A without and with inference performed on the edges using the simple bootstrap procedure, respectively. It is evident from the graphs that by performing no inference on the edges, many false positive edges are estimated. This simulation highlights the need for carrying out the bootstrap inferential procedure on the edges (section 2.5).

### 5.3. Simulation 3

**Figure 3:**
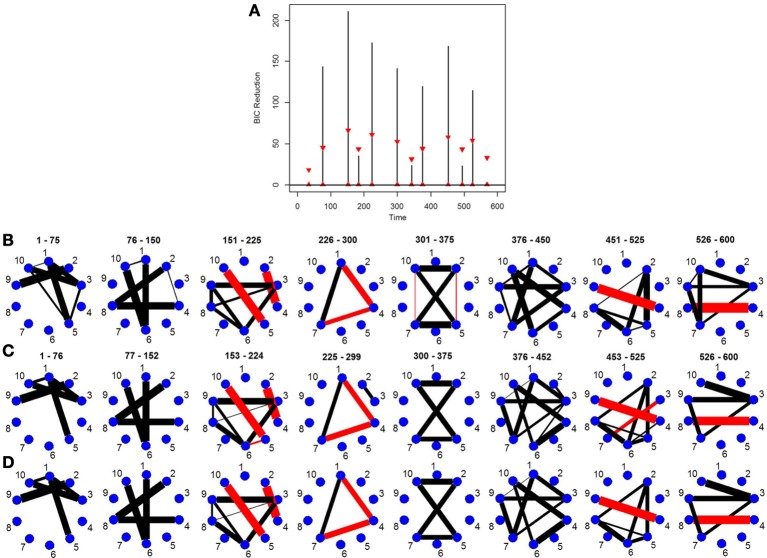
**(A)** The splitting times plotted against BIC reduction for the VAR data set with seven connectivity change points (simulation 3) using the new DCR algorithm. **(B)** The true undirected graphs for each partition. The undirected graphs for this data set using the new DCR and **(C)** no inference on the edges and **(D)** inference on the edges.

This simulation is very similar to simulation 2 except in this case the data was generated using a VAR model with seven change points. Figure 3A depicts the splitting times plotted against BIC reduction. The new DCR correctly finds all the seven connectivity change points. Figure 3B depicts the true undirected graphs. Figures 3C,D show the undirected graphs for the partitions found in Figure 3A without and with inference performed on the edges using the bootstrap procedure, respectively. Again by not performing inference on the edges, false positive edges are estimated.

### 5.4. Simulation 4

**Figure 4:**
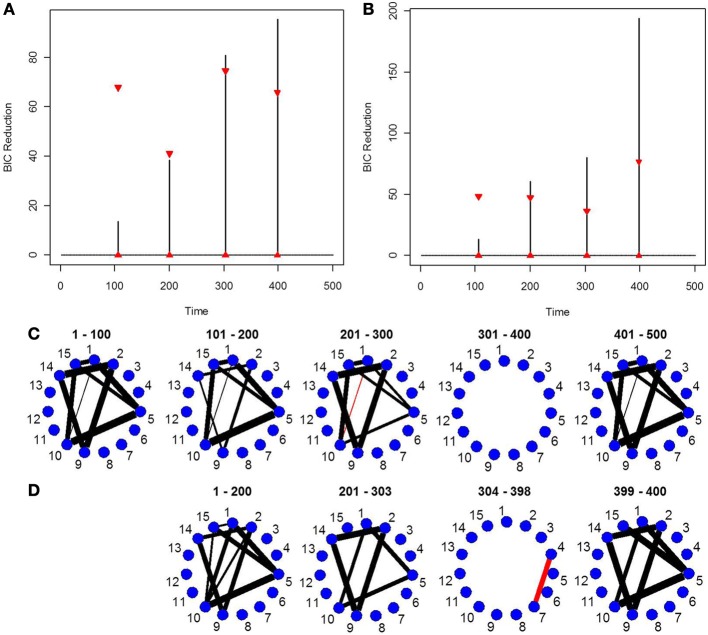
The splitting times plotted against BIC reduction for the VAR data set with four connectivity change points (simulation 4) using **(A)** the old DCR algorithm and **(B)** the new DCR algorithm. **(C)** The true undirected graphs for this data set and **(D)** the estimated undirected graphs for this data set using the new DCR.

This simulation shows how the new DCR performs in a weaker signal VAR environment. The true partial correlation structure is shown in Figure 4C. The only difference between Partition 1 (1–100 time points) and Partition 2 (101–200 time points) is that the magnitude of the partial correlation structure between brain regions 3, 6, and 9 is reduced while the correlation structure between 1, 4, 8, 14 remains the same. For Partition 3 (201–300 time points), the magnitude of the partial correlation structure between brain regions 1, 4, 8, 14 is reduced while the partial correlation structure between brain regions 3, 6, and 9 reverts back to the same correlation structure as in Partition 1. The connectivity structure in Partition 4 (301–400 time points) is i.i.d noise while Partition 5 (401–500 time points) has the exact same connectivity structure as Partition 1. This simulation depicts how the new DCR performs when the changes in connectivity are very small and in an ABA fMRI experimental setup.

The new DCR correctly identifies the correct change points but does not deem the change point at time point 100 to be significant (Figure 4B). Figure 4A shows the BIC reduction plotted against the splits using the old DCR algorithm. Notice the difference in magnitude of the BIC reduction and the stationary bootstrap confidence bounds. By recalculating the BIC reduction between change points a more accurate estimate is computed. The new DCR finds three of the four connectivity change points while the old DCR finds only two of the four. The new DCR method does a very good job of estimating the correct undirected graphs as well (Figure 4D). As the first change point is not deemed significant, the first estimated undirected graph is simply an average of the connectivity of the first two partitions.

### 5.5. Simulation 5

**Figure 5:**
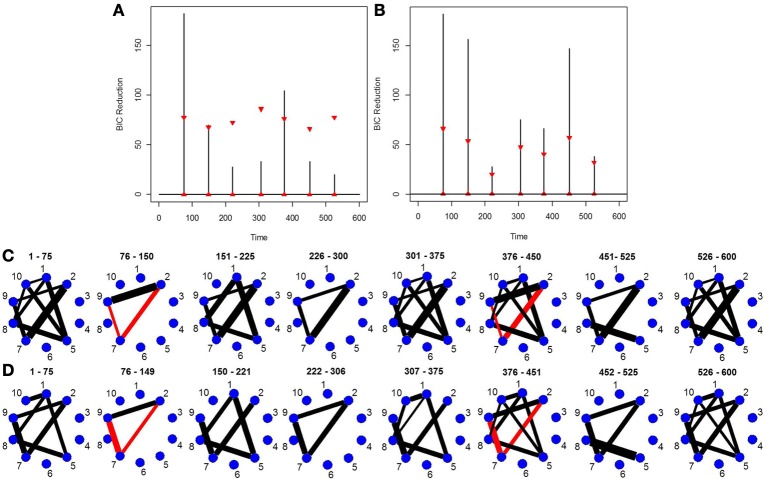
The splitting times plotted against BIC reduction for the VAR data set with seven connectivity change points (simulation 5) using **(A)** the old DCR algorithm and **(B)** the new DCR algorithm. **(C)** The true undirected graphs for this data set and **(D)** the estimated undirected graphs for this data set using the new DCR.

This simulation is similar to Simulation 4 in that the connected brain regions are constant for the entire time course but the magnitude and direction of the connectivity changes over time. This simulation contains more change points and more time points. Figure 5C shows the true undirected graphs for each partition. The new DCR correctly identifies all the change points (Figure 5B) and also performs very well in identifying the connectivity structure for all partitions (Figure 5D). Figure 5A shows the BIC reduction plotted against the splits using the old DCR. Notice the difference in magnitude of the BIC reduction and the stationary bootstrap confidence bounds. By recalculating the BIC reduction between change points a more accurate estimate is found. This simulation highlights the fact that the new DCR is very adept at locating small differences in the connectivity structure between two partitions for VAR data.

### 5.6. Simulation 6

**Figure 6:**
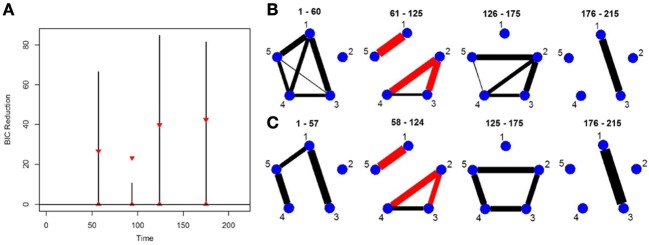
**(A)** The splitting times plotted against BIC reduction for the VAR data set (simulation 6) using the new DCR. The corresponding **(B)** true undirected graphs and **(C)** estimated undirected graphs for this data set.

This simulation is representative of the data in the real fMRI data that follows in the next section. It contains five brain regions and 215 time points and three connectivity change points. The true connectivity structure for this simulation can be seen in Figure 6B. The new DCR correctly identifies all the change points (Figure 6A) and also performs very well in identifying the connectivity structure for all partitions (Figure 6C). This simulation demonstrates how well the new DCR algorithm performs when the number of brain regions is small and the number of observations in the experimental time course is also small.

### 5.7. Simulation 7

**Figure 7:**
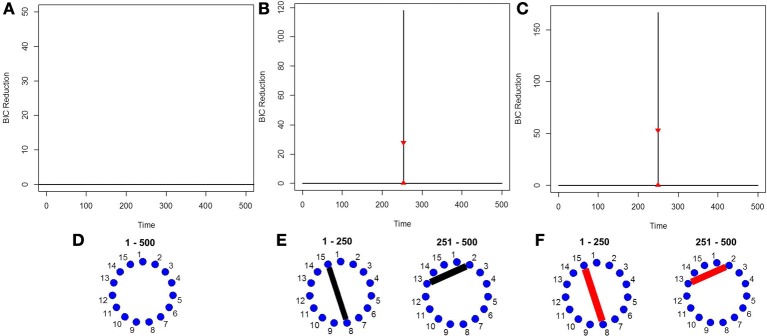
The splitting times plotted against BIC reduction for the VAR data set with one connectivity change point (simulation 7) using **(A)** the multi-subject DCR algorithm, **(B)** the new single-subject DCR (on one subject from group 1), **(C)** the new single-subject DCR (on one subject from group 2), and **(D–F)** their corresponding undirected graphs.

Finally, in order to show the importance of the new single-subject DCR methodology, we present an example where the multi-subject DCR algorithm (Cribben et al., 2012) fails to find the correct connectivity change points and the correct networks between brain regions but the new single-subject DCR discussed in this work performs very well. The setup of the simulation is as follows—we simulate a VAR model with 500 time points (*T* = 500) and 15 ROIs (*p* = 15) for 10 subjects, with the first five subjects having positive connectivity (average correlation ~0.67) between ROI 8 and 15 for the first 250 time points and positive connectivity (average correlation ~0.7) between ROI 2 and 13 for the final 250 time points. On the other hand, the second five subjects having negative connectivity (average correlation ~ −0.67) between ROI 8 and 15 for the first 250 time points and negative connectivity (average correlation ~ −0.7) between ROI 2 and 13 for the final 250 time points. The rest of the ROIs consist of i.i.d Gaussian noise. By running the multi-subject DCR algorithm (Cribben et al., 2012) on this data set and aggregating information across all 10 subjects, the two groups of subjects cancel each other out and no connectivity change points are found (Figure 7A) as well as no connectivity between ROI 8 and 15 or ROI 2 and 13 as well (Figure 7D). In this example, it is not a good idea to average the information across all 10 subjects.

Figures 7B,C show the BIC reduction plotted against the splitting time points using the new single-subject DCR algorithm for subject 1 (subject from group 1 which has positive connectivity between ROI 8 and 15 for the first 250 time points and positive connectivity between ROI 2 and 13 for the final 250 time points) and for subject 6 (subject from group 2 which has negative connectivity between ROI 8 and 15 for the first 250 time points and negative connectivity between ROI 2 and 13 for the final 250 time points), respectively. All subjects within group 1 and group 2 behave similarly. Note that by running the new DCR on the individual subjects we find the correct connectivity change points and the correct undirected graphs (Figures 7E,F). This simulation illustrates how vital it is to estimate connectivity changes for the individual subjects and to carry out the estimation as accurately as possible. After looking at the individual subjects, we can then make an informed decision on whether we should combine information across subjects and run the multi-subject DCR algorithm.

### 5.8. Experimental data

**Figure 8:**
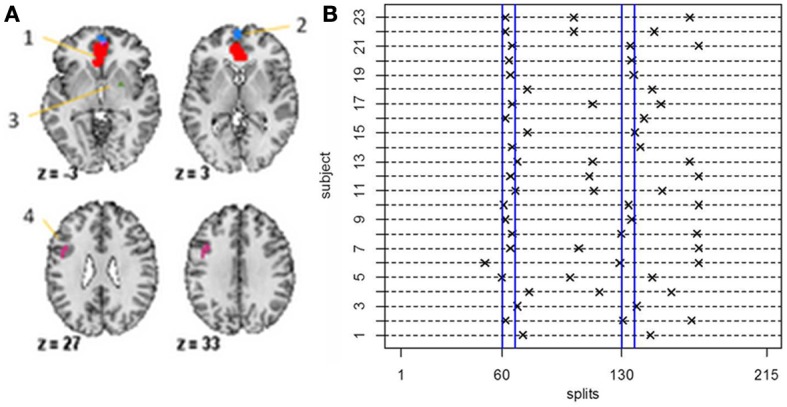
**(A)** The four-ROI and heart rate data set—the regions are: (1) VMPFC, (2) anterior mPFC, (3) Striatum/pallidum, and (4) DLPFC/IFJ. **(B)** The splitting times plotted against BIC reduction for the fMRI data set (four ROIs and heart rate) for all 23 subjects using the new DCR.

For the fMRI data set, in order to guarantee reasonable estimates of the sample mean vectors and sample covariance matrices, we set δ = 40, that is, we set the minimum distance between change points to be 40 data points. For the stationary bootstrap, we use ψ = 1/0.05, that is, the average block length is equal to 20 time points for a data set containing 100 time points. After conclusion of the algorithm, we plot the BIC reduction against time in order to find the significant change points. Figure 8B shows the significant change points for all subjects in the 4 ROIs and heart rate data set. The y-axis depicts the subject number while the x-axis shows the splitting times. Every subject has either two or three significant change points with each subject having a change point in the neighborhood of time point 60 (120 s), which corresponds directly to the presentation of the first visual cue specifying the topic of the speech and the removal of said cue at time point 67.5 (135 s). While all subjects have a significant splitting time in the vicinity of the first cue, this pattern does not continue across all subjects for the duration of the experiment. After this, the subjects split into two groups; group 1 (subjects 1, 3, 9, 14, 15, 16, 18, 19, 20) have only one further significant splitting time near time point 130 while group 2 (subjects 2, 4, 5, 6, 7, 8, 10, 11, 12, 13, 17, 21, 22, 23) have two further significant change points between the first cue and the end of the experiment. At time point 130 (260 s), the second visual cue stating that the participants would in fact not have to give the presentation to the expert panel of judges after the conclusion of the scanning session was revealed.

**Figure 9:**
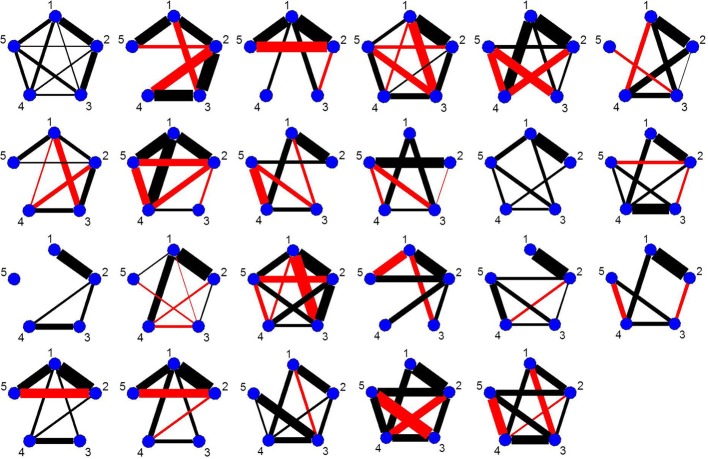
The undirected graphs for the first partition of all subjects in the four ROIs and heart rate data using the new DCR. The heart rate has label 5 in the undirected graphs.

As every subject has a significant change point in the vicinity of the first visual cue, Figure 9 shows the undirected graphs for the first partition for every subject corresponding to their individual change points in Figure 8B. The empirical quantile threshold for the bootstrap distribution of the edge values is 0.75 based on 1000 runs following evidence of stability from the simulations. The black and red edges in the undirected graphs represent positive and negative partial correlation, respectively. The thickness of the edges are directly related to the strength of the connectivity between two brain regions.

The connectivity structure in the undirected graphs for each subject is very different (Figure 9). The new bootstrap LR test discussed in section 2.6 was carried out in order to perform a test on the similarity of the precision matrices. The test statistic, Λ = 1860, results in a *p*-value of 0 rejecting the null hypothesis of equal precision matrices. However, the individual partial correlations shown in Figure 9 do have some overlap between subjects.

For subjects in Group 1, we also compared their precision matrices while the participants were silently preparing their speeches (time points 67.5–130) and their precision matrices while the subjects rested after they were informed that they would not have to deliver the speech. In each case, we rejected the null hypothesis (*p*-value = 0) of equal precision matrices implying significant heterogeneity in connectivity across subjects.

## 6. Discussion

DCR is an approach for splitting the experimental time course in a functional neuroimaging experiment into partitions based on functional connectivity changes between ROIs or voxels. In this work, we focus exclusively on the analysis of single-subject data, and extend the methodology in two ways: (1) we alter the algorithm in order to make the change point estimation more accurate with a small number of observations and (2) we perform inference on the edges in the undirected graph or connections between brain regions in order to reduce the number of false positive edges in the graphs. The new algorithm could be used for finding functional connectivity change points for multi-subject data as well. However, in was shown in Cribben et al. (2012) that the old DCR performed very well on multi-subject data and did not suffer from the issue of false positive edges in the undirected graphs to the same degree as the single-subject analysis.

DCR can be applied directly to data from ROI studies or to temporal components obtained from a PCA or ICA analysis. It is very flexible in that it does not require prior knowledge of the nature of the experimental design and may be particularly appropriate for studies when it is not possible to replicate experimental manipulations within subjects. For this reason, on going collaborations include applying the method to data of A-ha! or Eureka moments in order to observe the connectivity changes as the subject approaches the insight moment and where the state changes occured. It is also being applied to resting state data, pain data and data from hallucination experiments. We believe the introduction of the new DCR methodology for single-subject analysis as well as the new bootstrap Likelihood Ratio test for sparse precision matrices will be particularly beneficial to resting state data analysis as it is well known that there exists large heterogeneity in connectivity across subjects and between runs for the same subject.

The simulation study carried out indicates that the new DCR method is very capable of finding small and subtle changes in the connectivity structure as well as robust to the inclusion of spikes in that data. The simulations also show that the addition of weaker signals do not adversely affect the results.

In the original DCR paper (Cribben et al., 2012), the method was predominantly validated on multi-subject data by stacking subjects on top of one another in the algorithm. By combining information across subjects, we average the connectivity patterns in the group of subjects as they perform a set of tasks or change psychological state. Hence, we assume that they have the same connectivity structure. However, group average results may belie the unique activity patterns of individual subjects and by analysing individual subjects we may be able to determine if an individual scan is normative with respect to a reference population or we may be able to understand the sources of intersubject variability in brain activity (Van Horn et al., 2008). Simulation 7 illustrates the importance of the new single-subject DCR algorithm. By applying the multi-subject DCR algorithm to this data set, the first group of subjects with positive connectivity is negated by the second group of subjects with negative connectivity resulting in no connectivity change points being found and no significant networks being found in the undirected graphs. The correct connectivity change points were found by running the new single-subject DCR algorithm on the individual subjects. Hence, by looking at the individual subjects first we can make an informed decision about whether it is wise to run the multi-subject DCR algorithm and combine information across the group. This new single-subject allows us to perform this single-subject analysis accurately and reliably. Also, after performing the new DCR algorithm on the individual subjects from an fMRI study of state anxiety, we compared and tested the precision matrices or graphs across the individuals using a Likelihood Ratio test. Although the graph had some edges in common, we rejected the null hypothesis of equal precision matrices.

The choice of Δ in DCR is of particular importance as it represents the minimum possible number of time points between adjacent change points. Ideally, we want to make Δ as large as possible to ensure that there is enough data to provide reliable estimates of the sample means and sample covariance matrices. However, the choice of Δ places an upper bound on the number of change points that can be found using our method, with small values allowing for more change points. Hence, we want to make Δ as small as possible to ensure that we find all possible change points. Ultimately, its value can be adjusted depending on the existence of *a priori* knowledge about the spacing of changes in functional connectivity. For the multi-subject analyses we were able to choose a Δ of eight time points but for the single-subject analyses in this paper the smallest possible Δ was 30 time points. Hence, the single-subject analyses should only be applied to slowly changing state studies or where the distance between state changes was greater than 30 time points.

A common question asked about DCR is how long the computation takes and how many ROIs it can handle. It is difficult to estimate precisely given that the alogrithm depends on several interconnected variables. The method's computation time depends on the number of time points in the data set, the number of change points, the number of ROIs, the permutation or bootstrap procedures and the size of Δ. As the first four increase in number the computation time increases while as Δ decreases the computation time increases. Also, as the number of ROIs increase, interpretation of the undirected graphs becomes more difficult and the number of changes in the functional connectivity increases as well. The code is currently being converted to Matlab and paralellized in order to reduce the computational burden. We plan to make it available on our websites.

The permutation and stationary bootstrap inferential procedures for determining whether or not a change point was significant were reconfigured for use with the new single-subject DCR. In addition to inference on the change points, we also introduced a new bootstrap inference procedure for the edges or connections between brain regions in order to remedy the issue of excessive false positive edges in the estimation of undirected graphs after the change points had been found. The simulation study showed how vital this extra step is for the interpretation of the undirected graphs between each pair of change points.

In conclusion, the new single-subject DCR method, introduced in this paper, will be especially helpful and informative to people interested in functional connectivity changes in individuals and to determine variability across subjects. We are hopeful this is the beginning of further work in the identification of emotion, stress or resting state transitions and aberrant connectivity patterns and will help our understanding of the human brain and neurological disorders such as depression and Alzheimer's disease, and the treatment of these disorders.

### Conflict of interest statement

The authors declare that the research was conducted in the absence of any commercial or financial relationships that could be construed as a potential conflict of interest.

## A. Appendix

### A.1. New DCR algorithm

The algorithm for performing single-subject DCR is set up as follows [(7–10) are the extra steps]:

1. Consider the full data set. Calculate the sample mean and covariance matrix using Equations (1, 2), respectively. Obtain an estimate of Ω = Σ^−1^ for each value of λ using Equation (3).
2. Choose the value of λ and hence the estimate of Ω, that minimizes the BIC (Equation 4).
3. Refit the model without the lasso penalty term (i.e., the *l*_1_-constraint) keeping the zero elements in the matrix fixed and record the minimum BIC.
4. Partition the data into two parts: a left subset consisting of time points {1:γ} and a right subset consisting of {γ + 1:T} where γ = Δ. Repeat Steps (1–3) for both data sets and sum the BIC scores from the two partition elements. Repeat this procedure for γ values from Δ + 1 to *T* − Δ + 1.
5. If the sum of the combined BIC scores for any two subsets is less than the BIC of the entire data set computed in (2), the time point with the largest decrease is selected as the candidate change point, thus partitioning the data into two segments.
6. Apply Steps (1–5) recursively to each partition until no partition element can be further split into smaller elements.
7. Order the candidate change points once the algorithm is exhausted.
8. For the first candidate change point ρ, partition the data into two parts: a left subset consisting of time points {1:ρ} and a right subset consisting of {ρ + 1:ν} where ν represents the second candidate change point. Repeat Steps (1–3) for both subsets and sum the BIC scores from the two.
9. If the sum of the combined BIC scores for both subsets is less than the BIC of the entire data set consisting of time points {1:ν}, the time point remains a candidate change point.
10. Repeat steps (7–9) for all change points.

### A.2. The vector autoregression (VAR) model

The VAR model is one of the most used models for the analysis of multivariate time series models. It is used predominantly in the field of economics and finance to describe the dynamics of prices and markets. For a (*n* × 1) vector of time series variables **Y**_*t*_ = (*y*_1*t*_, *y*_2*t*_,…, *y_nt_*)^*T*^, the *p*-lag vector autoregressive (VAR(*p*)) model has the form

(A1) $$Y_{t}\text{​}=\text{​ }c+\Pi_{1}Y_{t-1}+\Pi_{2}Y_{t-2}+\ldots +\Pi_{p}Y_{t-p}+\epsilon_{t},t=1,\ldots ,T$$

where ∏_*i*_ are (*n* × *n*) coefficient matrices and ϵ_*t*_ is an (*n* × 1) unobservable mean white noise vector process with time invariant covariance matrix Σ (Zivot and Wang, 2003). For example, the equation for a univariate VAR(2) model is

(A2) $$\begin{array}{l} \left(\begin{array}{c} y_{1t}\\ y_{2t} \end{array}\right)=\left(\begin{array}{c} c_{1}\\ c_{2} \end{array}\right)+\left(\begin{array}{c} \pi_{11}^{1}\;\;\pi_{12}^{1}\\ \pi_{21}^{1}\;\;\pi_{22}^{1} \end{array}\right)\left(\begin{array}{c} y_{1t-1}\\ y_{2t-1} \end{array}\right)\\ \;+\left(\begin{array}{c} \pi_{11}^{2}\;\;\pi_{12}^{2}\;\\ \pi_{21}^{2}\;\;\pi_{22}^{2} \end{array}\right)\left(\begin{array}{c} y_{1t-2}\\ y_{2t-2} \end{array}\right)+\left(\begin{array}{c} \epsilon_{1t}\\ \epsilon_{2t} \end{array}\right) \end{array}$$

or

(A3) $$\begin{array}{l} y_{1t}=c_{1}+\pi_{11}^{1}y_{1t-1}+\pi_{12}^{1}y_{2t-1}+\pi_{11}^{2}y_{1t-2}+\pi_{12}^{2}y_{2t-2}+\epsilon_{1t}\\ y_{2t}=c_{2}+\pi_{21}^{1}y_{1t-1}+\pi_{22}^{1}y_{2t-1}+\pi_{11}^{2}y_{1t-2}+\pi_{22}^{2}y_{2t-2}+\epsilon_{2t} \end{array}$$

where *cov*(ϵ_1*t*_, ϵ_2*t*_) = σ_12_ for *t* = *s*; 0 otherwise.
